# An integrated view of lipid metabolism in ferroptosis revisited via lipidomic analysis

**DOI:** 10.1038/s12276-023-01077-y

**Published:** 2023-08-23

**Authors:** Jong Woo Kim, Ji-Yoon Lee, Mihee Oh, Eun-Woo Lee

**Affiliations:** 1 Metabolic Regulation Research Center, Korea Research Institute of Bioscience and Biotechnology (KRIBB), Daejeon, 34141 Korea; 2 Department of Functional Genomics, University of Science and Technology (UST), Daejeon, 34141 Korea; 3 Biodefense Research Center, Korea Research Institute of Bioscience and Biotechnology (KRIBB), Daejeon, 34141 Korea; 4 School of Pharmacy, Sungkyunkwan University, Suwon, 16419 Korea

**Keywords:** Metabolism, Cell death

## Abstract

Ferroptosis is a form of regulated cell death characterized by iron-dependent lipid peroxidation. This process contributes to cellular and tissue damage in various human diseases, such as cardiovascular diseases, neurodegeneration, liver disease, and cancer. Although polyunsaturated fatty acids (PUFAs) in membrane phospholipids are preferentially oxidized, saturated/monounsaturated fatty acids (SFAs/MUFAs) also influence lipid peroxidation and ferroptosis. In this review, we first explain how cells differentially synthesize SFA/MUFAs and PUFAs and how they control fatty acid pools via fatty acid uptake and β-oxidation, impacting ferroptosis. Furthermore, we discuss how fatty acids are stored in different lipids, such as diacyl or ether phospholipids with different head groups; triglycerides; and cholesterols. Moreover, we explain how these fatty acids are released from these molecules. In summary, we provide an integrated view of the diverse and dynamic metabolic processes in the context of ferroptosis by revisiting lipidomic studies. Thus, this review contributes to the development of therapeutic strategies for ferroptosis-related diseases.

## Unraveling ferroptosis: the iron-dependent cell death

A new study reveals the role of lipid metabolism in ferroptosis and its potential implications for cancer treatment. The researchers found that certain lipids, such as polyunsaturated fatty acids (PUFAs), are more susceptible to lipid peroxidation, leading to ferroptosis. By manipulating lipid metabolism pathways, the researchers were able to increase or decrease the sensitivity of cancer cells to ferroptosis, offering new therapeutic strategies for overcoming drug resistance and improving cancer treatment outcomes. Future research will focus on understanding the precise molecular mechanisms of ferroptosis and how they can be targeted for more effective cancer therapies.

## Introduction

**Fig. 1:**
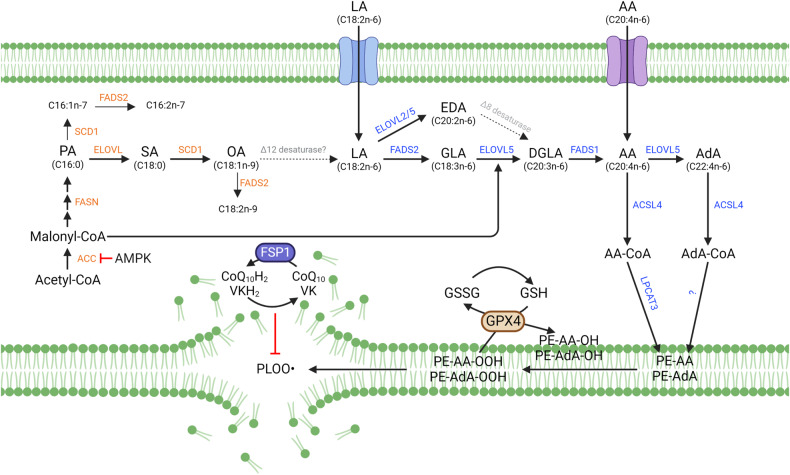
Fatty acid biosynthesis and ferroptosis. Arachidonic acid (AA, C20:4) is an n-6 polyunsaturated fatty acid that can be synthesized from linoleic acid (C18:2) by fatty acid desaturases (FADSs) and elongation of very long-chain fatty acid proteins (ELOVLs) or taken up directly from the environment. AA and its elongation product, adrenic acid (AdA), are incorporated into membrane phospholipids via acyl-CoA synthetase long-chain family member 4 (ACSL4) and lysophosphatidylcholine acyltransferase 3 (LPCAT3). PE-AA and PE-AdA are considered the most vulnerable phospholipids to peroxidation, which may be mediated by lipoxygenases or via nonenzymatic autoxidation reactions. Peroxidation is followed by the generation of lipid radicals, such as the phospholipid peroxyl radical (PLOO∙), which contributes to the lipid peroxidation chain reaction and, ultimately, to ferroptosis. Ferroptosis can be prevented by reducing lipid hydroperoxides to lipid alcohols via glutathione peroxidase 4 (GPX4) or by directly halting lipid radicals via ferroptosis suppressor protein (FSP1)/CoQ_10_/vitamin K (VK). The de novo lipogenesis (DNL) pathway contributes to the pool of saturated fatty acids (SFAs) and monounsaturated fatty acids (MUFAs). Although nonessential polyunsaturated fatty acids (PUFAs), such as n-7 and n-9 PUFAs, can be synthesized by FADS2 (Δ6 desaturase), mammals cannot synthesize essential PUFAs, such as n-3 and n-6 fatty acids, because they lack of Δ12 and Δ15 desaturases. Nonetheless, through the DNL pathway, SFAs, and MUFAs contribute to the abundance of n-6 PUFAs, positively or negatively impacting ferroptosis. Abbreviations: ACC acetyl-CoA carboxylase, AMPK AMP-activated protein kinase, FASN fatty acid synthase, GSH glutathione, GSSG glutathione disulfide, SCD1 stearoyl-CoA desaturase-1.

Ferroptosis is an iron-dependent, non-apoptotic form of cell death triggered by the lipid peroxidation of polyunsaturated fatty acids (PUFAs) in the cell membrane^1–3^. Lipids are continuously peroxidized through non-enzymatic autoxidation or lipoxygenase (LOX)-mediated lipid peroxidation under physiological conditions, particularly cellular stress conditions^4–12^. However, the generation of potentially deleterious lipid hydroperoxides is continuously surveilled by the selenoenzyme glutathione peroxidase 4 (GPX4); this enzyme is unique because it directly reduces lipid hydroperoxides in the acyl chains of phospholipids to lipid alcohols at the same time that reduced glutathione (GSH) is oxidized to form glutathione (GSSG) (Fig. 1)^8,13–15^. However, when GPX4 is inactivated or when GSH is depleted in cells, phospholipid peroxides (PLOOH) accumulate, leading to the generation of lipid radicals, such as lipid peroxyl radicals ((P)LOO∙) and alkyl radicals ((P)L∙), through iron-dependent free radical chain reactions^10,16,17^. Uncontrolled lipid peroxidation and the generation of reactive aldehydes secondary to lipid peroxidation ultimately cause cell membrane rupture and cell death via ferroptosis (Fig. 1)^1,2,18–20^. Notably, endogenous lipophilic radical trapping agents (RTAs), such as coenzyme Q10 and vitamins E and K, can halt the lipid peroxidation chain reaction, thereby functioning as non-enzymatic defense mechanisms against ferroptosis (Fig. 1)^8,10^. Moreover, the reduction of oxidized RTAs via ferroptosis suppressor protein 1 (FSP1), previously known as apoptosis-inducing factor mitochondria-associated 2 (AIFM2), is essential for the protective effect of RTAs against ferroptosis (Fig. 1)^17,21–28^.

While lipid radicals are the primary factors driving ferroptosis, their sources remain unclear. Mitochondria are considered the most prominent generators of reactive oxygen species (ROS) in a cell due to incomplete reduction of molecular oxygen; thus, mitochondrial ROS are required for ferroptosis^29^. Notably, inhibiting the electron transport chain, which can generate superoxide anion (O_2_^−^∙) by releasing electrons, blocks ferroptosis induced by GSH depletion^30^. Superoxide is mainly deposited in the mitochondrial matrix and generated in the intermembrane space via complex III, and superoxide can be exported from mitochondria to the cytoplasm via voltage-dependent anion channels. However, the precise mechanisms by which mitochondrial superoxide is released into the cytoplasm or at a membrane to induce lipid peroxidation remain unclear^31,32^. In addition to mitochondrial ROS, glutaminolysis and the tricarboxylic cycle (TCA) affect the ferroptosis rate^33^. Inhibition of oxidative phosphorylation by deleting cytochrome c oxidase assembly factor 10 causes lysosomal and mitochondrial defects, resulting in the lipid peroxidation and ferroptosis of cardiac cells^34^. Furthermore, mitochondria are dispensable for ferroptosis induced by GPX4 inhibition because GPX4 rapidly amplifies the small number of lipid peroxides already present in the cell membrane^30^. Although the mitochondria-independent pathway driven by GPX4 inhibitors is crucial for understanding and improving cancer treatment, -dependent ferroptosis induced by cysteine deficiency or GSH depletion may be more relevant to the pathophysiological context of chronic human diseases.

PUFAs in membrane phospholipids are sensitive to lipid peroxidation and have been repeatedly associated with ferroptosis^35^. Thus, with a focus on lipidomic studies, this review summarizes the different mechanisms by which cells maintain the pool of PUFAs; control their incorporation into specific phospholipids, such as a diacyl or ether lipid with a specific phospho-head group; undergo lipid peroxidation, and cause ferroptosis. We also discuss how the levels of saturated fatty acids (SFAs), monounsaturated fatty acids (MUFAs), and PUFAs are mutually regulated, stored in lipid droplets, and recycled.

## Identification of pro-ferroptotic PUFAs

Because lipid peroxidation is a hallmark of ferroptosis, researchers have wondered whether specific lipids cause ferroptosis^1,4,5,36,37^. When ferroptosis is induced by piperazine erastin, a more stable analog of erastin, phosphatidylcholine (PC) species containing PUFAs are depleted in cells, while the levels of lysophosphatidylcholine (LPC) species and ceramide are increased^2^. This outcome suggests that PC-PUFAs are preferentially oxidized, followed by their degradation or phospholipase-mediated cleavage, increasing the levels of LPCs. A direct analysis of oxidized lipids in GPX4-knockout (KO) cells revealed that hundreds of phospholipids are oxidized during ferroptosis. This finding suggests that lipid peroxidation in membranes can readily extend among membrane phospholipids via a lipid peroxidation chain reaction, eventually causing cellular membrane disruption^38^. Kagan et al. analyzed oxidized phospholipids that were significantly correlated with cell death. Those authors identified di- and tri-oxygenated phosphatidylethanolamines (PEs) containing arachidonic acid (AA) and adrenic acid (AdA) chains, namely, PE-C18:0/C20:4 and PE-C18:0/C22:4, as ferroptotic signaling lipids (Fig. 1)^38^. RSL3, a GPX4 inhibitor, markedly increased the levels of oxygenated PE species, similar to the effect of GPX4 deficiency, suggesting that these PE species are common ferroptotic signaling molecules^38^. Based on these findings, subsequent studies supported the supposition that oxidized PE-AA and PE-AdA are ferroptosis- withaferin A promoting lipids^7,11,39–42^.

Independent studies have shown that phospholipids in addition to PEs are oxidized via ferroptotic stimuli. Treatment of bone marrow-derived macrophages with ML162, a GPX4 inhibitor, increased the amounts of oxidized phospholipids, such as PE, phosphatidylserine (PS), and phosphatidylinositol (PI)^43^. Furthermore, the levels of di- and tetra-oxygenated phosphatidylglycerol (PG) and PI species were increased in neuroblastoma tumor cells treated with withaferin A, a phytochemical that induces ferroptosis^16^. Notably, Withaferin A treatment also increased the levels of mono-oxygenated (+1°) PE and PC species, which may be products of lipids peroxidized via the lipid peroxidase activity of GPX4^16^. Furthermore, RSL3 treatment markedly increased the amount of oxidized PE and ether-linked PE (ePE) species. Specifically, PE plasmalogens (PE-p) carry various long-chain PUFAs, such as AA (C20:4) and docosapentaenoic acid (DPA, C22:5). The establishment of these oxidized PUFAs was completely prevented by vitamin K, recently discovered to be a natural ferroptosis inhibitor^27^. Liver tissues from hepatocyte-specific *Gpx4*-KO mice exhibited robust oxidation of various phospholipids, including PE and PE-p, which carry various PUFA chains^27^. The amounts of oxidized PEs and PE-p containing an oleic acid (OA, C18:1) increased in RSL3-treated or *GPX4*-deleted cells^27^. OA suppressed ferroptosis; however, whether oxidized PE-OA plays a specific role in ferroptosis or is simply oxidized during a non-enzymatic free radical chain reaction remains unclear^10^. Thus, whether most lipids are oxidized in response to ferroptotic stimuli is unclear. Notably, only specific lipids may foster early peroxidation events followed by non-specific oxidation of other lipids.

## Phospholipid remodeling by the Lands cycle in ferroptosis

**Fig. 2:**
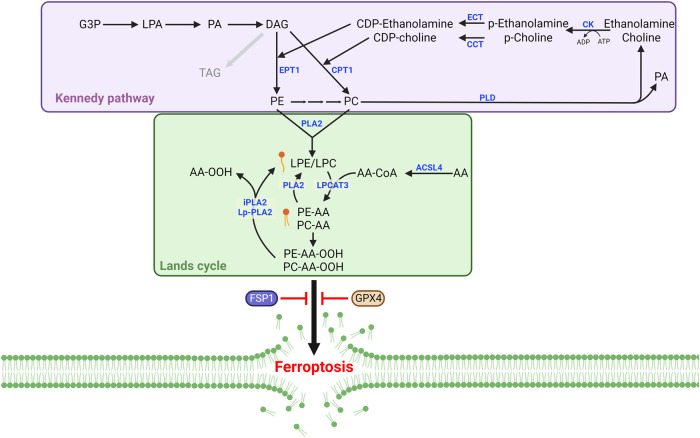
The Kennedy pathway and Lands cycle in ferroptosis. The Kennedy pathway is critical for de novo phosphatidylethanolamine (PE) and phosphatidylcholine (PC) synthesis. Phospholipase A2 (PLA2) enzymes, including cytosolic PLA2 (cPLA2), calcium-independent phospholipase A2 (iPLA2), and lipoprotein-associated PLA2 (Lp-PLA2), cleave the *sn-2* position of phospholipids, contributing to lysophosphatidylethanolamine (LPE) and lysophosphatidylcholine (LPC) recycling. The *sn-2* position of phospholipids favors PUFAs, and PLA2 enzymes release PUFAs, such as AA and oxidized AA, from phospholipids. Abbreviations: CCT choline-phosphate cytidylyltransferase, CK choline kinase, CPT1 choline phosphotransferase 1, DAG diacylglycerol, ECT ethanolamine-phosphate cytidylyltransferase, EPT1 ethanolaminephosphotransferase 1, G3P glycerol-3-phosphate, LPA lysophosphatidic acid, PA phosphatidic acid, PLD phospholipase D.

Cells can directly synthesize PE and PC species via the Kennedy pathway and synthesize these phospholipids via the Lands cycle from lysophosphatidylethanolamine (LPE), LPCs, and free fatty acids (FFAs) (Fig. 2)^44–47^. AA, possibly the most relevant PUFA involved in ferroptosis, is largely absent in plants; notably, mammals obtain AA primarily from consuming meat, including that of fish^48–50^. Extracellular AA, which can enter the cell directly in an orchestrated manner, is incorporated into the phospholipids of cellular membranes via a series of enzymatic reactions^46,47,51^. The first step of phospholipid synthesis is the acylation of fatty acids, through which coenzyme A (CoA) is ligated to fatty acids by acyl-CoA synthetase long-chain family member 4 (ACSL4) with high preference for AA (Figs. 1, 2)^52^. Arachidonoyl-CoA (AA-CoA) is then esterified in membranes by lysophosphatidylcholine acyltransferase 3 (LPCAT3), which preferentially binds LPC and LPE to produce membrane phospholipids in the endoplasmic reticulum (ER) (Figs. 1, 2)^53^. Global lipidomic analysis using *LPCAT3*-KO embryos confirmed that the levels of membrane phospholipids, especially PE or PC containing AA are generally reduced in the absence of LPCAT3^54^. Notably, through genome-wide screening approaches, ACSL4 and LPCAT3 have been identified as genes critical to ferroptosis, suggesting that PE-AA is the most relevant phospholipid involved in ferroptosis^55,56^.

Numerous independent studies have supported a critical role for ACSL4 in ferroptosis^55,57,58^. A recent study suggested that ACSL4 was required for GPX4 inhibitor-induced ferroptosis but not for erastin or cysteine deprivation-induced ferroptosis^59^. These findings suggest that slower progression of ferroptosis induced by cysteine deficiency may bypass PE-AA-dependent lipid peroxidation. Several studies have also shown that LPCAT3 plays a vital role in ferroptosis^38,56,60,61^. Specifically, inhibiting LPCAT3 suppressed ferroptosis, although not completely^61^. Moreover, inhibiting LPCAT3 activity may cause an accumulation of AA-CoA, which is then elongated by ELOVL5 to produce AdA-CoA, allowing the production of PE-AdA (C22:4) independent of LPCAT3 action^61,62^. Similar findings have been obtained in *LPCAT3*-KO mice, which presented with higher amounts of C22 or C24 PUFA chains in PEs and PCs, possibly because of the elongation and desaturation of free AA (C20:4) followed by its esterification into phospholipids independent of LPCAT3^54^.

Phospholipids are rapidly deacylated and reacylated in cells via the Lands cycle (Fig. 2). PUFAs, such as AA, are typically anchored to the *sn-2* position of phospholipids and can be deacylated via the phospholipase A2 (PLA2) family, which liberates AA from phospholipids^63,64^. Therefore, PE-AA abundance may determine ferroptosis sensitivity. Nonetheless, direct evidence of PLA2 family enzymes controlling PE-AA levels and playing an important role in ferroptosis remains scarce. iPLA2β, encoded by *PLA2G6*, can cleave peroxidized PE-AA, also known as 15-hydroperoxy-eicosatetraenoyl-phosphatidylethanolamine (15-HpETE-PE), removing the toxic lipid peroxide and protecting cells against ferroptosis^39,65,66^. Notably, human placentas from spontaneous preterm births accumulated oxidized PE species, including PE(38:4) + 3[O] and PE(36:4) + 2[O], implying the involvement of ferroptosis in this process. Further research revealed that iPLA2β plays a role in placental ferroptosis. Additionally, a lack of *PLA2G6* expression increased fetal mortality following RSL3 treatment by facilitating ferroptosis^39^. iPLA2β also protects against p53-mediated ferroptosis after ROS stimulation, thereby contributing to the tumor-suppressive role of p53^65^. In addition to iPLA2β, peroxiredoxin 6 (PRDX6), which also shows iPLA2 activity, suppresses ferroptosis, although lipidomic alterations were not explicitly evaluated in this study^67^.

Lipoprotein-associated PLA2 (Lp-PLA2), encoded by *PLA2G7*, preferentially cleaves oxidized PC in low-density lipoproteins (oxLDLs), producing oxidized nonesterified fatty acids (oxNEFAs) and LPC, which contribute to the progression of atherosclerosis^68^. Lp-PLA2 cleaves oxidized PE-AA, 1-steaoryl-2-15-HpETE-*sn*-glycero-3-phosphatidylethanolamine (SAPE-OOH) in a manner analogous to that of iPLA2^69^. However, in contrast to iPLA2β, extracellular Lp-PLA2 cleaves SAPE-OOH from the phospholipids in the outer bilayer of the plasma membrane, which protects ferroptotic cells from macrophage-mediated phagocytosis^69^. However, whether Lp-PLA2 contributes to ferroptosis remains unclear. Lp-PLA2 is also found inside cells and protects them against ferroptosis by downregulating PE species by cleaving oxidized AA^70^. Thus, deletion or inhibition of Lp-PLA2 results in the accumulation of PE species, including PE(38:4), facilitating ferroptosis^70^. This lipidomic shift occurs after 1 h of treatment with darapladib, an inhibitor of Lp-PLA2, indicating that the Lands cycle is rapidly progressing in cells. The Lands cycle may function with greater specificity than the Kennedy pathway on PUFAs, such as AA, to induce the lipid rewiring that affects ferroptosis susceptibility. Considering that the Lands cycle fundamentally regulates phospholipid levels and is swiftly and actively running in the cells^71^, identifying how, when, and where ferroptotic phospholipids levels are controlled is essential for understanding ferroptosis regulation.

## The n-6 PUFA biosynthesis pathway and ferroptosis

Cells can directly take up AA (C20:4n-6) from their environment, and linolenic acid (LA, C18:2n-6), a precursor of AA and an omega-6 essential fatty acid, is much more abundant in human blood, implying that cells may need to synthesize AA directly from LA (Fig. 1)^72–75^. Using ^13^C-labeled LA, we recently showed that cancer cells can directly synthesize AA from LA^62^. The elongation of very long-chain fatty acid protein 5 (ELOVL5) and fatty acid desaturase 1 (FADS1) are involved in PUFA synthesis enzymes. Furthermore, these enzymes have been shown to play essential roles in ferroptosis in mesenchymal-type gastric cancer cells (Fig. 1)^62^. Intestinal-type gastric cancer cells fail to express ELOVL5 and FADS1 after their promoters are methylated, rendering cells resistant to ferroptosis induced via GPX4 inhibition or cysteine depletion^62^. In another context, FADS2, rather than FADS1, increases the sensitivity of hepatocarcinoma cells to ferroptosis^76,77^. Lipidomic analyses showed that depletion of either FADS1 or FADS2 through siRNA transfection successfully reduced AA levels, although only FADS2 depletion suppressed ferroptosis. The accumulation of dihomo-γ-linolenic acid (DGLA, C20:3) after FADS1 depletion might contribute to ferroptosis, as DGLA undergoes lipid peroxidation in certain contexts (Fig. 1)^76,78^. In addition, FADS2-mediated ferroptosis inhibited hepatitis C virus (HCV) replication, and triple-negative breast cancer cells with high FADS1/2 expression were found to be more susceptible to ferroptosis^76,79^.

**Fig. 3:**
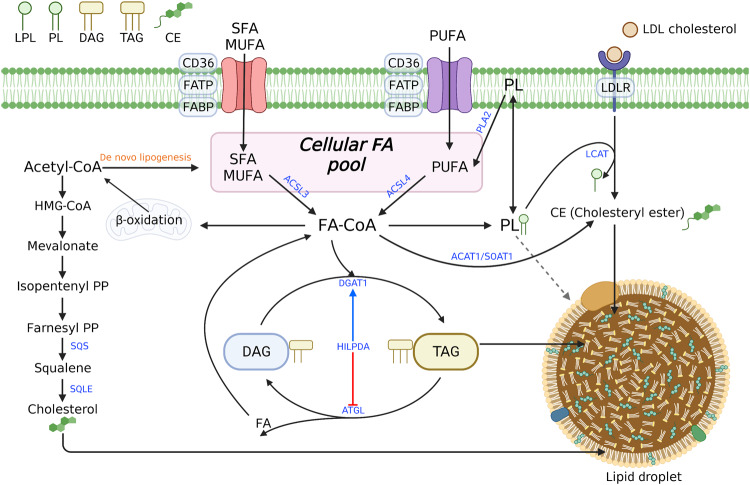
Integrated view of the metabolism of fatty acids and various lipids. The DNL pathways and exogenous supply determine the abundance of fatty acids through the fatty acid transporters CD36 and FATP and the LDL receptor (LDLR). Free fatty acids can be stored in neutral lipids such as triacylglycerols (TAGs), cholesteryl esters (CEs), and phospholipids. Several enzymes release fatty acids from these stores, resulting in either lipid remodeling or degradation via β-oxidation. Although PUFAs in phospholipids are regarded as pro-ferroptotic lipids, the roles of the remodeling pathways of these lipids in ferroptosis are still unclear. ACSL enzymes that catalyze fatty acyl-CoA synthesis play various roles, including fatty acid oxidation, phospholipid synthesis, TAG synthesis, and CE synthesis. Abbreviations: ACAT1 acyl-coenzyme A cholesterol acyltransferase 1, ACSL acyl-CoA synthetase long-chain, ATGL adipose triglyceride lipase, DGAT1 diacylglycerol O-acyltransferase 1, DNL de novo lipogenesis farnesyl, PP farnesyl pyrophosphate, HILPDA hypoxia-inducible lipid droplet associated, isopentenyl PP isopentenyl pyrophosphate, LCAT lecithin–cholesterol acyltransferase, SOAT1 sterol O-acyltransferase 1.

Activation of LA (C18:2) via ACSL1 is required for subsequent PUFA synthesis and β-oxidation, suggesting that ACSL1 plays a crucial role in ferroptosis in certain contexts (Fig. 3)^80,81^. However, *ACSL1*-KO BT549 cells were not protected against ML162-induced ferroptosis^82^. Further research should be focused on clarifying whether BT549 cells preferentially synthesize AA endogenously or whether their uptake of AA is facilitated by the absence of *ACSL1*. Moreover, cancer cells with high PUFA synthesis activities depend on ACSL4 for RSL3-induced ferroptosis. The proportion of AA that is imported into a cell is low compared with the total AA level, and the internalized AA is primarily stored in the triglycerides of lipid droplets rather than as membrane phospholipid chains^83^. Therefore, both synthesis and import pathways may be required to determines the total AA pool. Moreover, the reincorporation of AA into phospholipids via ACSL4 is likely to be crucial for lipid peroxidation and associated ferroptosis.

ELOVL5, which mediates the elongation of AA into AdA, may play a pivotal role in ferroptosis; however, the impact of AdA on ferroptosis has not been clearly characterized (Fig. 1). Gastric cancer cells with *ELOVL5*-KO completely lost the ability to synthesize AA from LA. Nonetheless, the total amounts of AA and PE-AA were marginally decreased and in some cases were even increased in these cells, which may be ascribed to the compensatory effects of cell culture medium^62^. However, AdA and PE-AdA levels were markedly reduced in *ELOVL5*-KO cells because serum contains only low AdA levels^72–74^. The resistance of *ELOVL5*-KO cells to ferroptosis suggested that PE-AdA plays a critical role in ferroptosis. This finding is supported by several lipidomic studies showing that AdA levels are more substantially altered than AA levels^38,55,62^.

## De novo lipogenesis and energy metabolism in ferroptosis

Ferroptosis is governed by various metabolic mechanisms involved in lipid, iron, and redox metabolism. Therefore, several studies have examined the interplay between the ferroptosis and other metabolic pathways^35,84–87^. The energy stress driven by glucose depletion suppressed ferroptosis via AMP-activated protein kinase (AMPK) activation^88^. Then, activated AMPK phosphorylated and inhibited acetyl-CoA carboxylase (ACC) action, suppressing the de novo synthesis of fatty acids such as palmitate (Fig. 1)^88^. Lipid profiling revealed that the amounts of MUFAs and PUFAs also decreased when cells are treated with the AMPK activator A769662 or ACC inhibitor TOFA^88^. In contrast, the quantity of these lipids was generally higher in *AMPKα1/α2* double KO (DKO) ACHN cells than in wild-type cells^88^. Thus, AMPK-mediated energy metabolism may govern the abundance of pro-ferroptotic PE-PUFAs and function as a critical regulator of ferroptosis^88^. Similarly, glucose deficiency suppressed ferroptosis by inhibiting glycolysis, the TCA cycle, and fatty acid synthesis mediated via pyruvate dehydrogenase kinase 4 (PDK4)^89^. In cells treated with erastin, glucose depletion led to a decreased level of most PE species regardless of their fatty acid chains, possibly reducing the fatty acid biosynthesis^89^.

Mammals lack the Δ12 desaturase and cannot desaturate OA (C18:1n-9) into LA (C18:2n-6), an essential fatty acid. Therefore, how the de novo lipogenesis pathway and palmitate abundance cause n-6 PUFA accumulation remains unclear^48,90,91^. ACC-dependent production of malonyl-CoA may facilitate PUFA biosynthesis, but LA uptake may also accompany de novo lipogenesis to efficiently increase PUFA abundance (Fig. 1). Interestingly, although AMPK activation leads to a reduction in the levels of most lipid classes, including lysoPLs, ACC inhibition by TOFA induces the accumulation of lysoPLs and downregulated production of FFA and PLs. These findings imply that ACC affects PLA2 activity and that AMPK plays an additional role in lipid remodeling independent of ACC action (Figs. 1, 2)^88^.

The effect of de novo lipogenesis on lipid remodeling has also been studied using *KRAS*-mutant lung cancer cells after fatty acid synthase (FASN) inhibition. In contrast to ACC inhibition, FASN inhibition via TVB-3166 induced ferroptosis in the absence of ferroptosis inducers by upregulating PCs containing PUFAs while downregulating PCs with SFA and MUFA chains^60^. Although FASN inhibition can result in the accumulation of malonyl-CoA, a metabolic flux analysis using ethyl acetate-2-^13^C revealed that the direct elongation of LA (C18:2) to AA (C20:4) was not facilitated, suggesting that AA may be imported from the microenvironment^60^. Considering that LPCs generally accumulate in TVB-3166-treated cells, a previous study proposed a combination model in which the Lands cycle and de novo pathways are activated in *KRAS*-mutant cells^60^. Thus, LPCs are continuously produced via the Lands cycle, and the SFAs and MUFAs which synthesized via the de novo pathway are incorporated into phospholipids (Fig. 2). FASN inhibition decreased the levels of SFAs and MUFAs while relatively increasing the levels of PUFAs, resulting in the accumulation of PC-PUFAs^60^. In addition, total LPC levels were increased, while total PC levels remained unchanged. The Kennedy pathway may also be accelerated to compensate for the inhibition of fatty acid synthesis^60^.

Treatment of the LNCaP-LN3 prostate cancer cell line with the FASN inhibitor TVB-3166 decreased palmitate content, resulting in the accumulation of total lipids, whereas supplementation with palmitate reversed the acquisition of this phenotype^92^. In contrast to that in *KRAS*-mutant lung cancer cells, the increase in total lipids in prostate cancer cells was not due to an increase in fatty acid uptake because similar lipidomic changes were observed under serum-free conditions^92^. A lipidomic analysis showed that several lipids, such as PE, PC, and LPC, accumulated after FASN inhibition, implying that the Kennedy pathway was activated to compensate for palmitate depletion^92^. The amounts of PUFAs, including AA, also increased, but the underlying mechanisms remain unknown^92^. Collectively, the de novo fatty acid synthesis pathway is closely associated with other lipid metabolizing processes, such as PUFA and phospholipid metabolism. However, the inhibition of ACC1 and FASN exerted opposite effects on PUFA abundance, thereby differentially regulating ferroptosis. An integrated study covering various lipid metabolism pathways is required to understand lipidome dynamics and how they relate to ferroptosis-related diseases.

## MUFAs and ferroptosis

Generally, MUFAs, such as palmitoleic acid (C16:1n-7) and OA (C18:1n-9), are synthesized from SFAs by stearoyl-CoA desaturase 1 (SCD1). MUFA levels are determined mainly by SCD1, the rate-limiting enzyme in MUFA synthesis, although further desaturation and elongation into n-7 and n-9 PUFAs can occur in mammalian cells^91^. MUFAs, which exhibit antioxidant properties, strongly inhibit ferroptosis^2,75,93,94^. A lipidomic analysis suggested that OA reduced the amounts of ferroptosis-related phospholipids, such as PE or PC containing AA or AdA. OA likely competes with AA and AdA for incorporation into phospholipids. Nonetheless, the number of PCs or PEs containing OA is unchanged when cells are treated with supplemental OA despite the subsequent increase in free OA. This suggests that a more complex mechanism is involved^94^. In addition, OA did not inhibit ferroptosis in *ACSL3*-depleted cells, as ACSL3 mediates the incorporation of MUFAs into phospholipids, suggesting that MUFA incorporation into phospholipids is crucial for protection against ferroptosis^94,95^. Considering the increase in the OA-containing PI level after OA supplementation, PI may also impact ferroptosis sensitization^94^. Although this study focused on phospholipid synthesis involving ACSL3, esterification of ACSL3 was shown to be a critical step for β-oxidation and lipid droplet formation, which may contribute to ferroptosis vulnerability (Fig. 3).

Melanoma cells from lymph fluid are much more resistant to ferroptosis than those from blood plasma, rendering melanoma metastasis via the lymph much more efficient^95^. Lymph contains a large amount of OA, mostly in the form of triacylglycerols (TAGs) in ApoB^+^ vesicles, which transport circulating lipids into cells^95,96^. Although OA protects melanoma cells against ferroptosis, *ACSL3*-deleted melanoma cells undergo ferroptosis even in the presence of OA, supporting a critical role for ACSL3 in MUFA-mediated ferroptosis suppression^95^.

Recently, membrane-bound O-acyltransferase 2 (MBOAT2), also known as LPCAT4, was identified as a GPX4/FSP1-independent ferroptosis suppressor via two-independent clustered regularly interspaced short palindromic repeats (CRISPR) screening assays^97,98^. Previous studies had shown that MBOAT2 has preference for OA. Similarly, a significant increase was observed in PEs and ePEs with C16:1 or C18:1 chain in cells overexpressing MBOAT2^53,97^. In contrast, the levels of pro-ferroptotic PEs and ePEs with various PUFA chains were markedly reduced in MBOAT2-overexpressing cells, leading to ferroptosis resistance^97^.

SCD1 is a critical regulator of MUFA synthesis, and its expression contributes to ferroptosis resistance in several types of cancer cells by increasing MUFA levels^99–101^. Cell lines harboring a *PIK3CA*-activating mutation or a *PTEN*-inactivating mutation showed ferroptosis resistance mediated via activation of the PI3K/AKT/mTOR pathway, which further upregulates SREBP1^101^. Among various SREBP1 targets, SCD1 is the most significantly reduced by *SREBP1* deletion in cancer cells, suggesting that SCD1-mediated MUFA synthesis is a key driver of ferroptosis resistance^101^. SCD1 is also upregulated reoxygenation after hypoxia during tumor recurrence, thereby protecting cancer cells against ferroptosis and increasing cancer cell survival^100^. Notably, vitamin B12 deficiency in *Caenorhabditis elegans* abrogated the methionine cycle, resulting in the SREBP1-dependent expression of *fat-6* and *fat-7*, both of which encode SCD^102^. *C. elegans* harbors Δ12 desaturase, but vitamin B12 deficiency caused the accumulation of n-6 PUFAs and MUFAs, leading to ferroptosis and infertility in *C. elegans*^102^.

## Fatty acid uptake and ferroptosis

A common mechanism underlying malignant and chemo-resistant tumor formation is altered lipid metabolism^51,85,86,103,104^. These type of tumor cells are frequently susceptible to ferroptosis, for which various underlying mechanisms have been proposed^62,105^. Androgen-targeted therapy with enzalutamide of LNCaP PCa cells increased the total lipid levels, including those of several phospholipids containing PUFAs^106^. Enzalutamide treatment generally downregulates the expression of enzymes involved in PUFA synthesis while substantially upregulating that of lipid transporters, such as the LDL receptor (LDLR) and scavenger receptor type B1 (SCARB1). Therefore, this treatment may activate lipid uptake^106^. In contrast, this treatment significantly downregulates the expression of cholesteryl esters (CEs) and TAGs by inhibiting the de novo synthesis of cholesterol and fatty acids, as demonstrated by isotope-tracing experiments and the downregulating effects of SREBF2 and ACLY expression^106^. Enzalutamide-treated cells were more prone to ferroptosis than control cells; thus, ferroptosis sensitivity may be determined by the abundance of PUFA-containing phospholipids not by that of other lipids.

Cells are supplemented with exogenous fatty acids via fatty acid transport proteins, such as fatty acid translocase (FAT/CD36), fatty acid transport protein (FATP), fatty acid-binding protein (FABP), and LDLR (Fig. 3). Nonetheless, the precise role of these proteins in ferroptosis remains unclear^51,104,107–109^. Increased expression of the CD36 fatty acid transporter in tumor-infiltrating CD8^+^ T cells has been associated with poor clinical outcomes in patients receiving PD-1 inhibitor therapy^110,111^. Notably, the absence of CD36 in CD8^+^ T cells isolated from mice markedly altered the expression of genes related to lipid metabolism, resulting in ferroptosis resistance due to a reduced lipid peroxidation^111^. Exogenous AA uptake via CD36 was required for ferroptosis in tumor-infiltrating CD8^+^ T cells and resulted in the loss of anti-tumor immunity. Thus, inhibiting ferroptosis via the T-cell specific deletion of *CD36* may enhance anti-PD-1-mediated immunotherapy^111^. In addition, oxygenated lipids such as 1-stearoyl-2-15(S)-HpETE-*sn*-glycero-3-PE/PC (PE/PC-18:0/20:4-OOH) were released by myeloid-derived suppressor cells (MDSCs), which are neutrophils that are activated during ferroptosis^112^. These lipids were required for the immunosuppressive function of MDSCs; thus, inhibiting ferroptosis exerted a beneficial effect during immunotherapy by protecting neutrophils against ferroptosis^112^. In contrast, tumor cells from mice treated with anti-PD-L1 present with high lipid peroxidation rates, and ferroptosis inhibition via liproxstatin-1 treatment abolished the mouse response to anti-PD-L1 and anti-CTLA combination therapy^113^. Notably, the AA level in tumor interstitial fluids is higher than that in peripheral blood, and AA in the tumor microenvironment is primarily incorporated into PE via ACSL4, as evidenced by AA-d5 tracing experiments^58^. The mechanism by which AA uptake by cancer cells is facilitated by CD8^+^ T cells remains unclear. Nonetheless, AA supplementation significantly increased anti-PD-L1 anti-tumor immunity in immunocompromised mice^58^. TYRO3, a receptor tyrosine kinase expressed in cancer cells, suppressed T-cell mediated ferroptosis by upregulating NRF2 pathway activation^114^. Inhibition of TYRO3 enhanced cancer immunotherapy by inducing tumor ferroptosis and altering the M1/M2 macrophage ratio^114^. Therefore, whether ferroptosis is beneficial for cancer immunotherapy remains unclear because of the contrasting results obtained to date immune checkpoint inhibitors have been used^84,115,116^.

## Lipid droplets and ferroptosis

Although de novo fatty acid synthesis and uptake from the microenvironment are crucial in establishing the pool of fatty acids in cells, the regulation of the levels of fatty acids stored as TAGs in lipid droplets and their subsequently release in response to stimuli are critical for cellular function (Fig. 3)^117,118^. TAGs may be an important PUFA reservoir, contributing to ferroptosis by supplying PUFAs for incorporation into phospholipids. Through genome-wide CRISPR screening, several HIF pathway genes were shown to be essential for ferroptosis in clear-cell renal cell carcinoma (ccRCC) cells. These genes included *EPAS1*, encoding HIF2α, and *ARNT*, encoding HIF-1β^119^. A lipidomic analysis of 786-O cells with *EPAS1* deleted revealed that the number of TAGs and other phospholipids were generally reduced in *EPAS1*-KO cells^119^. Although the amounts of free SFAs and MUFAs are also reduced in *EPAS1*-KO cells, the reduction in the levels of free PUFAs was more pronounced and caused decreased amounts of PEs containing PUFAs^119^. A screening assay with cells overexpressing each of the HIF2 target genes revealed that hypoxia-inducible lipid droplet-associated protein (HILPDA) and G0/G1 Switch 2 (G0S2) re-sensitized *EPAS1*-KO cells to ferroptosis (Fig. 3)^119^. A lipidomic analysis showed that G0S2 increased the total TAG amount, whereas HILPDA increased the number of TAGs containing PUFAs^119^. PUFAs anchored to TAGs were directly oxidized and degraded during ferroptosis, as imidazole ketone erastin (IKE) treatment eliminated TAG-PUFAs as well as PCs and PEs with PUFA chains^120^. G0S2 and HILPDA ultimately cause the accumulation of PEs containing PUFAs. Nevertheless, whether PUFAs bound to TAGs are important for direct peroxidation or whether PUFAs are transferred to phospholipids in the context of ferroptosis remains unclear.

Because ACSL-mediated fatty acid activation contributes to TAG formation, ACSL proteins can regulate ferroptosis. HIF1/2 activation in VHL-deficient ccRCC cells caused lipid droplet accumulation in an ACSL3-dependent manner^121^. Cells exposed to charcoal-stripped FBS lost lipid droplets, suggesting fatty acid transport is mediated by HIF1/2^121^. Moreover, *ACSL3* deletion prevented erastin-induced ferroptosis possibly by reducing the TAG levels in lipid droplets^121^. The mechanism by which ACSL3 deficiency affects ferroptosis warrants further analysis considering that AA incorporation into phospholipids is primarily activated by ACSL4 but not by ACSL3^121^. Furthermore, this finding contradicted a previous study suggesting that inhibiting ACSL3 facilitated ferroptosis by reducing the availability of MUFAs, which are potent suppressors of ferroptosis^94^.

In contrast to their positive role in ferroptosis, lipid droplets can prevent ferroptosis. Excessive amounts of n-3 and n-6 PUFAs triggered ferroptosis under acidosis, which mimics tumor microenvironment conditions^122^. Cancer cells may escape ferroptosis by storing toxic PUFAs in TAGs. Thus, they might inhibit diacylglycerol O-acyltransferase (DGAT) function, which is required for TAG synthesis, which results in the accumulation of PUFAs in phospholipids to reach high levels enhance ferroptosis^122^.

## Cholesterol and ferroptosis

Although cholesterol is a vital lipid and is susceptible to oxidation, its function in ferroptosis remains unclear^117,123,124^. However, intermediate metabolites of the mevalonate pathway clearly play an important role in ferroptosis (Fig. 3)^51,125,126^. In brief, isopentenyl-pyrophosphate (IPP) is required for the synthesis of selenoproteins, including GPX4, and the isopentenylation of selenocysteine-tRNA stabilizes selenocysteine-specific tRNAs, contributing to ferroptosis suppression^105,127^. In addition, farnesyl-pyrophosphate (FPP) is a precursor of ubiquinone or coenzyme Q10 (CoQ_10_), a strong inhibitor of ferroptosis^21,25,128,129^. Squalene epoxidase (SQLE), the rate-limiting enzyme in cholesterol biosynthesis, may play a role in ferroptosis because squalene accumulation in cholesterol auxotrophic lymphomas caused by SQLE loss suppressed ferroptosis^130^. Circulating tumor cells derived from patients with melanoma showed resistance to ferroptosis and upregulated expression of SREBP2, which preferentially transactivates genes in the cholesterol biosynthesis pathway^131^. SREBP2 directly mediates the expression of transferrin, which may sequester intracellular iron, thereby preventing lipid peroxidation and ferroptosis^131^.

Treatment with high doses of 27-hydroxycholesterol (27-HC), a common type of circulating cholesterol, inhibited cancer cell proliferation, but chronic treatment led to cancer cell resistance to treatment by upregulating lipid uptake^129^. Notably, 27-HC-resistant cancer cells acquire ferroptosis resistance, leading to increased tumorigenicity and more metastatic properties, although how these lipids specifically inhibit ferroptosis is unclear^129^. Furthermore, SCARB1 is a receptor of high-density lipoproteins (HDLs) enriched with cholesterol. Therefore, targeting this protein using HDL-like nanoparticles (HDL-NPs) blocked cholesterol uptake but induced a compensatory response that leads to the de novo synthesis of cholesterol^132^. HDL-NPs downregulated GPX4 mRNA expression, thereby directly inducing ferroptosis, although the underlying mechanisms remain unknown^132^.

In an attempt to identify phospholipid transporters. such as scramblases, flippases, and floppases, the lipid flippase solute carrier family 47 member 1 (SLC47A1) was identified as an anti-ferroptotic protein^57^. *SLC47A1*-depleted cells exhibited showed increased levels of PUFA-containing CEs that were produced via acyl-CoA cholesterol acyltransferase 1 (ACAT1), also known as sterol O-acyltransferase 1 (SOAT1)^57^. The levels of both PEs and PCs containing PUFAs remained unchanged when SLC47A1 was depleted; however, CE-PUFAs were more prone than PE-PUFAs to lipid peroxidation, suggesting that the flippase function of SLC47A1 may contribute to ferroptosis^57^.

## Ether phospholipids and ferroptosis

**Fig. 4:**
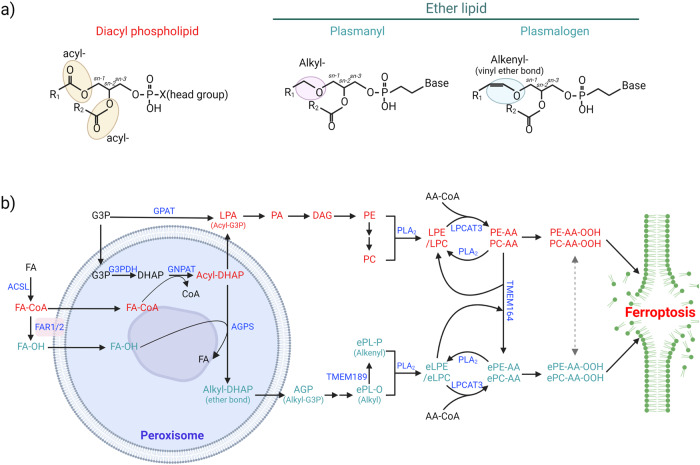
Schematic showing the diacyl and ether phospholipid synthesis pathways during ferroptosis. **a** Diacyl phospholipids contain two fatty acyl chains, each linked with an ester bond, whereas ether lipids consist of a fatty alkyl chain connected by an ether bond or a fatty alkenyl chain connected by a vinyl ether bond at the *sn-1* position. **b** Diacyl and ether lipid synthesis mechanisms. Lysophosphatidic acid (LPA) can be synthesized directly from glycerol-3-phosphate (G3P) in the ER or mitochondria by glycerol-3-phosphate acyltransferase (GPAT) or indirectly from G3P by acyl-dihydroxyacetone phosphate (acyl-DHAP) in peroxisomes, followed by diacyl phospholipid synthesis. The peroxisomal enzymes fatty acyl-CoA reductases (FAR1/2) generate fatty alcohol (FA-OH), which is then replaced with alkyl-DHAP via alkylglycerone phosphate synthase (AGPS), resulting in ether lipid synthesis. Alkenyl-ether lipids, known as plasmalogens, are synthesized from alkyl-ether lipids by TMEM189. ePE-AA and ePC-AA can be synthesized from eLPE, eLPC, and AA-CoA by LPCAT family enzymes or from PC-AA by TMEM164, which transfers the AA chain from PC-AA to an eLPE or eLPC. Although both diacyl- and ether phospholipids containing PUFAs are prone to lipid peroxidation, their relative contributions to initial lipid peroxidation and ferroptosis may be context dependent. Abbreviations: G3PDH glyceraldehyde 3-phosphate dehydrogenase, GNPAT glyceronephosphate O-acyltransferase, PA phosphatidic acid, alkyl-DHAP alkyl-dihydroxyacetone phosphate, TMEM164 transmembrane protein 164, TMEM189 transmembrane protein 189, AGP 1-*O*-Alky glycerol-3-phosphate.

Diacyl phospholipids have typically received most of the attention in ferroptosis research; however, recent research has revealed that PUFA-containing ether phospholipids (ePLs) with an alkyl-ether or vinyl ether bond at the *sn-1* position may play important roles in determining ferroptosis sensitivity (Fig. 4a)^133,134^. In an initial study with researchers using genome-wide CRISPR‒Cas9 suppressor screening, genes involved in peroxisome biogenesis, such as peroxisomal biogenesis factor 10 (*PEX10*) and peroxisomal biogenesis factor 3 (*PEX3*), as well as those encoding peroxisomal enzymes, such as alkylglycerone phosphate synthase (*AGPS*) and fatty acyl-CoA reductase 1 (*FAR1*), were identified (Fig. 4b)^133,134^. Considering the importance of peroxisomes in ether phospholipid synthesis, lipidomic analysis was performed with two cancer cell lines, the OVCAR-8 and 786-O cell lines, in which the expression of *PEX10* or *FAR1* had been abrogated. The authors found that *PEX10* or *FAR1* deficiency reduced the amounts of various ether lipids, primarily ether-phosphatidylethanolamine (ePE) and ether-phosphatidylcholine (ePC) containing PUFAs, resulting in ferroptosis resistance^133,134^.

The inactivation of peroxisome genes specifically reduced the number of ePLs containing PUFAs; however, the specific mechanisms that preferentially mediate PUFA abundance in ether phospholipids are unknown^133^. In addition, transmembrane protein 164 (TMEM164) has been recently discovered to transfer AA directly from PCs to lyso-ePEs, resulting in a specific increase in the ePE-AA level (Fig. 4b)^135,136^. TMEM164 has also been suggested to induce autophagy, resulting in ferritin, GPX4, and lipid droplet degradation^136^. These findings suggest that TMEM164 is a crucial player in ferroptosis, and its inhibition is a novel strategy for inhibiting ferroptosis.

Given the indispensable role of ether phospholipids in ferroptosis, it is unclear whether ether lipids are more relevant than diacyl phospholipids initially triggering ferroptosis because both diacyl and ether phospholipids can increase ferroptosis and are eventually oxidized during ferroptosis (Fig. 4b)^27,133^. Researchers using murine small-cell lung cancer cells that spontaneously transdifferentiated from a non-neuroendocrine (non-NE) state into a neuroendocrine (NE) state discovered that the cells in the non-NE state were ferroptosis sensitive^137^. Intriguingly, the diacyl PE-38:4 and PC-36:4 levels are lower in the ferroptosis-sensitive cells (non-NE) than in the ferroptosis-resistant cells (NE)^137^. However, neither ePEs or ePCs containing PUFAs were enriched in non-NE cells in which ether lipid synthesis enzymes were upregulated, implying the importance of ether lipids in ferroptosis^137^. In addition, deleting a-synuclein or *ACSL4* in LUHMES cells, neuronal precursor cells, resulted in a robust reduction in ePE and ePC levels regardless of fatty acid chain species^138^. Despite the slight increase in PUFA-containing diacyl PEs, a-synuclein- or *ACSL4*-depleted cells were resistant to ferroptosis, suggesting that ether lipids are more critical than diacyl lipids to ferroptosis, at least in this cellular context^138^.

Furthermore, targeting TMEM164, which specifically generates ePE-AA, reduced the oxidized form but not the unoxidized form of diacyl PE-AA, implying that ePE oxidation affects PE-AA oxidation to induce marked lipid peroxidation and ferroptosis^136^. TMEM164 may play an important role only in certain cellular contexts because ferroptosis resistance after *TMEM164* deletion has been associated with an increase in MUFA-containing ePEs, while the ferroptosis rates in cells with other types of phospholipid species were unaffected by *TMEM164* deficiency, as indicated because they do not show an accumulation of MUFA-ePEs^136^. In summary, the types of fatty acids attached to ePLs, rather than the ePLs alone, may determine ferroptosis sensitivity.

In addition, ether lipids, such as plasmalogens, exhibit antioxidant properties mediated through vinyl ether bonds, and plasmalogen-deficient cells were found to be more susceptible to oxidative stress^139^. *C. elegans* lacking the *ads-1* gene, encoding AGPS, or human HT-1080 cells treated with an AGPS inhibitor showed increased lipid peroxidation and ferroptosis rates^139^. Interestingly, out of 24 genetic screens aimed to identify essential genes in ferroptosis, *AGPS* was discovered in only five of them, implying that ether lipids may be context-dependent regulators of ferroptosis^59^. Although *AGPS* disruption successfully decreased the levels of ePLs containing PUFAs in HT-1080 cells, it increased the levels of several diacyl PE species, such as PE-16:0/22:4 and PE-16:0/22:4, resulting in these cells showing similar ferroptosis sensitivity similar to that of control cells^59^. Collectively, these data suggest that total PUFA levels or the relative composition of PUFAs may be more relevant than those of SFAs and MUFAs in determining the resistance or sensitivity of cells toward ferroptosis.

## Ferroptosis in human diseases

Several genetic and pharmacological studies have suggested that ferroptosis may be crucial to various human diseases^140^. GPX4 is an essential enzyme for embryonic development, as mice lacking *Gpx4* die by E7.5^18^. An initial study suggested that neuron-specific *Gpx4* depletion caused neurodegeneration, suggesting the possible involvement of ferroptosis in various neurodegenerative diseases, such as Alzheimer’s disease (AD) and Parkinson’s disease (PD)^19^. Iron-overload is a hallmark of many neurogenerative diseases, including AD, and iron levels have been associated with AD progression in clinical AD samples. Therefore, an iron chelator, deferiprone, is currently being evaluated in phase II clinical trials for patients with AD^141–144^. In addition, critical roles for ferroptosis in the underlying mechanisms of PD have been suggested in numerous studies^138,145,146^.

The role of ferroptosis in acute kidney injury (AKI) was first discovered in *GPX4*-knockout model mice. Inducible deletion of *GPX4* led to mouse death within a few weeks due to acute renal failure, which was markedly delayed in mice treated with liproxstatin-1, a ferroptosis inhibitor, and lipophilic RTA^20^. In addition, ferrostatin-1, a ferroptosis inhibitor, and its derivatives prolonged survival in model mice with renal ischemia‒reperfusion (I/R) injury^87,147^. Furthermore, mice deficient in *ACSL4*, a critical gene to ferroptosis, were resistant to I/R-induced AKI, supporting a critical role for ferroptosis in AKI^148^.

I/R-induced heart injury was also relieved by deferoxamine (DFO), an iron chelator^33^. In addition, doxorubicin-induced cardiomyopathy and I/R injury were prevented by ferrostatin-1 or dexrazoxane (DXZ), an FDA-approved iron chelator^149^. Increased expression of heme oxygenase-1 (HMOX-1) and downregulation of GPX4 have been suggested to contribute to cardiomyopathy^149,150^. Moreover, several studies have suggested a critical role for ferroptosis in various cardiovascular diseases, such as diabetic and septic cardiomyopathy, as well as atherosclerosis^151–153^.

## Concluding remarks

The fundamental processes of ferroptosis, such as lipid peroxidation and GPX4/GSH system functions, have been studied for a long time^15,19,154^. Nonetheless, ferroptosis has only recently (approximately 10 years) been characterized^1^. Moreover, an increasing body of research has revealed the molecular mechanism of ferroptosis and its relevance to various human diseases^114,118,150,155–158^. Despite extensive studies, the precise molecular mechanisms of ferroptosis still need to be identified because the relevant signaling pathways have not been clearly established, and only a few key players have been identified. Although the peroxidation of PUFAs in phospholipids in response to ferroptotic stimuli amplifies the number of lipid radicals produced via free radical chain reactions, ultimately leading to cell death, it is possible that antioxidants and membrane repair systems are also activated to resist ferroptosis^159–163^. The levels of several molecules critical to ferroptosis, such as GSH and CoQ10, are controlled by various metabolic pathways; therefore, for the accurate characterization of ferroptosis, metabolomic and lipidomic studies, which remain highly technically challenging, are needed^118,164–167^.

Several studies have suggested that certain metabolic states determine cell vulnerability to ferroptosis^35,84–86,103,118,162,168,169^. However, some studies have focused on a specific metabolic pathway of interest without considering overall metabolic changes. Therefore, despite the limited number of studies on metabolism with respect to ferroptosis, this review attempted to understand how various metabolic pathways are intertwined to regulate ferroptosis by focusing on lipidomic studies because several metabolic enzymes are associated with multiple metabolic pathways. For example, ACSL3 can activate different fatty acids, such as palmitate and OA, inducing their incorporation into phospholipids and TAGs, as well as β-oxidation in mitochondria (Fig. 3). Other molecular players, including kinases such as AMPK and transcription factors such as HIF, are also involved in various important signaling pathways that may affect cell sensitivity to ferroptosis independent of their roles in lipid metabolism. Therefore, the results described for each study must be carefully interpreted, as some conflicting results may be context dependent.

With increasing interest in the specific roles of distinct cellular compartments and organelles that impact ferroptosis, an important question remains about which membrane(s) initiate lipid peroxidation and how these lipid-derived oxidative signals can be ultimately transmitted to the cell membrane to disrupt it and subsequently induced necrotic cell death^47^. Although mitochondria may play context-dependent roles in ferroptosis, several studies have suggested that the ER is the most relevant organelle in ferroptosis initiation^83^. Notwithstanding, several studies have shown that lipid peroxidation occurs in the plasma membrane, which is crucial for eventual cell death^21,25^.

Although ferroptosis has been implicated in various human diseases, the mechanisms by which it is triggered in the context of these disease remain unknown. The critical roles of ferroptosis in ischemia–reperfusion injury in the liver, heart, and kidney, however, have been extensively characterized because the excessive supply of oxygen after ischemia results in the generation of ROS, such as superoxide, peroxyl radical, H_2_O_2_, and hydroxyl radicals, which promote lipid peroxidation and ferroptosis^34,87,149,155^. However, reoxygenation after oxygen-glucose deprivation does not specifically induce ferroptosis, suggesting other modes of induction. Nevertheless, ferroptosis inhibitors exert beneficial effects on several disease models, suggesting that ferroptosis is a critical checkpoint in disease progression and not a consequence of irreversible damage.

Moreover, ferroptosis induction is an interesting cancer therapy strategy because a single ferroptosis-inducing agent can effectively kill cancer cells, which is important considering the development of chemoresistance^84,87,115,116,118,170–172^. Although ferroptosis contributes to immunotherapy, inhibiting it may contribute to cancer immunotherapy effectiveness under specific circumstances, as T cells and neutrophils are sensitive to ferroptosis^84,111,112,115,116^. Thus, ferroptosis modulation in the context of immuno-oncology may function in a Janus-like fashion by either triggering cell death in cancer cells or maintaining tumor-directed immune cells in a fully functional state. In conclusion, establishing the precise role of ferroptosis in a specific disease context via a thorough understanding of the metabolic pathways is vital to treating a wide range of ferroptosis-related diseases.
